# The efficacy of the food-grade antimicrobial xanthorrhizol against *Staphylococcus aureus* is associated with McsL channel expression

**DOI:** 10.3389/fmicb.2024.1439009

**Published:** 2024-07-03

**Authors:** Elena A. Mordukhova, Jongwan Kim, Haiyan Jin, Kyoung Tai No, Jae-Gu Pan

**Affiliations:** ^1^GenoFocus Ltd., Daejeon, Republic of Korea; ^2^Bioinformatics and Molecular Design Research Center (BMDRC), Incheon, Republic of Korea; ^3^The Interdisciplinary Graduate Program in Integrative Biotechnology and Translational Medicine, Yonsei University, Incheon, Republic of Korea; ^4^Department of Biotechnology, Yonsei University, Seoul, Republic of Korea; ^5^Infectious Disease Research Center, Korea Research Institute of Bioscience and Biotechnology (KRIBB), Daejeon, Republic of Korea

**Keywords:** food-grade antimicrobial, xanthorrhizol, MscL, *Staphylococcus aureus*, mutants

## Abstract

**Background:**

The emergence and spread of multidrug-resistant *Staphylococcus aureus* strains demonstrates the urgent need for new antimicrobials. Xanthorrhizol, a plant-derived sesquiterpenoid compound, has a rapid killing effect on methicillin-susceptible strains and methicillin-resistant strains of *S. aureus* achieving the complete killing of staphylococcal cells within 2 min using 64 μg/mL xanthorrhizol. However, the mechanism of its action is not yet fully understood.

**Methods:**

The *S. aureus* cells treated with xanthorrhizol were studied using optical diffraction tomography. Activity of xanthorrhizol against the wild-type and *mscL* null mutant of *S. aureus* ATCC 29213 strain was evaluated in the time-kill assay. Molecular docking was conducted to predict the binding of xanthorrhizol to the SaMscL protein.

**Results:**

Xanthorrhizol treatment of *S. aureus* cells revealed a decrease in cell volume, dry weight, and refractive index (RI), indicating efflux of the cell cytoplasm, which is consistent with the spontaneous activation of the mechanosensitive MscL channel. *S. aureus* ATCC 29213Δ*mscL* was significantly more resistant to xanthorrhizol than was the wild-type strain. Xanthorrhizol had an enhanced inhibitory effect on the growth and viability of exponentially growing *S. aureus* ATCC 29213Δ*mscL* cells overexpressing the SaMscL protein and led to a noticeable decrease in their viability in the stationary growth phase. The amino acid residues F5, V14, M23, A79, and V84 were predicted to be the residues of the binding pocket for xanthorrhizol. We also showed that xanthorrhizol increased the efflux of solutes such as K^+^ and glutamate from *S. aureus* ATCC 29213Δ*mscL* cells overexpressing SaMscL. Xanthorrhizol enhanced the antibacterial activity of the antibiotic dihydrostreptomycin, which targets the MscL protein.

**Conclusion:**

Our findings indicate that xanthorrhizol targets the SaMscL protein in *S. aureus* cells and may have important implications for the development of a safe antimicrobial agent.

## Introduction

The sesquiterpenoid compound xanthorrhizol, isolated from the rhizomes of *Curcuma xanthorrhiza* Roxb. (Zingiberaceae), exhibits broad-spectrum antibacterial activity, mainly against gram-positive bacteria such as *S. aureus, Streptococcus mutans*, *Actinomyces viscosus*, *Porphyromonas gingivalis*, *Bacillus cereus*, *Listeria monocytogenes*, and *Streptococcus sali*var*ius* (Hwang et al., 2000a; Lee et al., 2008); against the yeast *Candida albicans* (Hwang et al., 2000b); and against various filamentous fungi (Rukayadi and Hwang, 2007). Xanthorrhizol has shown limited activity against gram-negative species (Mata et al., 2001; Lee et al., 2008; Oon et al., 2015). Mutation of the *imp* (*lptD*) gene significantly increased the susceptibility of *E. coli* cells to xanthorrhizol (Mata et al., 2001), which made it possible to identify two potential targets for xanthorrhizol in *E. coli*: tRNA-specific adenosine deaminase (TadA) and enoyl-ACP reductase (FabI) (Yogiara et al., 2015, 2020). In addition to its antimicrobial activity, xanthorrhizol also exhibits anticancer, anti-inflammatory, antioxidant and other biological activities without detectable toxic side effects (Choi et al., 2005; Chung et al., 2007; Oon et al., 2015). An inhibitory effect of xanthorrhizol was detected against severe acute respiratory syndrome (SARS) coronavirus 2 (SCoV2), as well as against other related coronaviruses such as SARS-CoV-1 (SCoV1) and the human coronavirus that causes the common cold (Kim et al., 2021).

A time-killing kinetic study showed that after 4 h of incubation with 8 μg/mL xanthorrhizol (1xMIC), no viable *S. aureus* cells were found (Lee et al., 2008). This rapid killing activity of xanthorrhizol has previously been shown against *S. mutans* (Hwang et al., 2000a), and electron microscopy analysis revealed disintegration of *S. mutans* cells, indicating that xanthorrhizol disrupted membrane structure or induced cell rupture (Kim et al., 2008). This effect is similar to that of osmotic downward shock, where the cell membrane ruptures, releasing intracellular content and resulting in cell death due to the large difference in osmotic strength between the cell and the environment (Chure et al., 2018). In response to changes in membrane tension and to avoid cell membrane damage, regulated efflux of water and small intracellular molecules occurs though transmembrane protein channels (Martinac et al., 1987; Chure et al., 2018). However, mechanosensitive channels not only protect bacteria from osmotic forces and prevent the selective release of cytoplasmic proteins under conditions of hypoosmotic shock (Kouwen et al., 2009a) but are also thought to play a role in the uptake and efflux of antibiotics (Iscla et al., 2014; Wray et al., 2016).

The *S. aureus* has the mechanosensitive channel of large conductance MscL (Liu et al., 2009; Dorwart et al., 2010) and one MscS type transporter, homologous to both *ykuT/mscT* (34.30%) and *yfkC/mscC* (35.50%) mechanosensitive channels of small conductance in *B. subtilis* (Hoffmann et al., 2008; Casey and Sleator, 2021). This surprisingly low presence of mechanosensitive channel nomenclature in *S. aureus* might be explained by the fact that hypoosmotic shock occurs relatively rarely in such an ecological niche as human skin (Casey and Sleator, 2021). The mechanosensitive channel MscL seems to be a very attractive target for antibiotic therapy due to its presence in almost all bacterial species but not in mammals, and it is highly conserved (Iscla et al., 2015). Some compounds have been shown to target mechanosensitive channel MscL in *S. aureus* (SaMscL) (Kouwen et al., 2009b; Iscla et al., 2015; Wray et al., 2019b, 2020, 2021, 2022; Blount and Iscla, 2020; Wang and Blount, 2022).

In the present study, we found that the natural antimicrobial agent xanthorrhizol acts by targeting the SaMscL mechanosensitive channel to inhibit the exponential growth of *S. aureus* cells, reduce the viability of *S. aureus* stationary cultures in a MscL-dependent manner, and enhance the anti-MscL activity of the antibiotic dihydrostreptomycin (Wray et al., 2016), which allows the use of xanthorrhizol as a potential antimicrobial agent.

## Materials and methods

### Bacterial strains, media, and culture conditions

The strains and plasmids used in this study are listed in Table 1. The LB medium for *E. coli* and tryptic soy broth (TSB) for *S. aureus* strain cultivation were purchased from Difco (San Jose, USA). The following concentrations of antibiotics were used: ampicillin, 100 μg/mL; erythromycin, 20 μg/mL; and kanamycin, 25 μg/mL. Dihydrostreptomycin sulfate (Sigma Aldrich St. Louis, MO) was used at concentrations ranging from 8 to 50 μg/mL. Xanthorrhizol was purchased from Cayman Chemical (Ann Arbor, Michigan, United States), and purified xanthorrhizol was also provided by Professor Jae-Kwan Hwang (Department of Biotechnology, Yonsei University, Seoul, Republic of Korea).

**Table 1:** Strains and plasmids used in this study.

Strain or plasmid	Relevant description	Source or reference
***E. coli***		
DH5α	*F-,supE44 hsdR17 recA1 gyrA96* *endA1 thi-1 relA1 deoR λ-*	Sambrook et al. (1989)
ET12567	*dam-13::Tn9 dcm-6 hsdM CmR*	New England Biolabs
***S. aureus***		
33592	Gentamicin and methicillin resistant	ATCC
29213	Wild-type	ATCC
29213Δ*mscL*	29213Δ*mscL* gene was deleted	This study
**Plasmids**		
pHoss1	Amp^R^, Ery^R^, pMAD::secY antisense, Δ*bgaB*^a^	Abdelhamed et al. (2015)
pHoss1/Δ*mscL*	Suicide plasmid for *mscL* gene deletion in *S. aureus* ATCC 29213, Amp^R^, Ery^R^	This study
pBE-S	Secretion vector, Amp^R^, Kan^R^, PaprE, SPaprE	Takara Bio Inc.
pHT01	Expression vector, Amp^R^, Cm^R^	MoBiTec GmbH
pBEP	pBE-S carrying the lacI gene and IPTG-inducible Pgrac promoter from pHT01, expression vector, Amp^R^, Kan^R^	This study
pBEP-SaMscL	Expression vector carrying the *mscL* gene from *S. aureus* ATCC 29213, Amp^R^, Kan^R^	This study

a Amp^R^, ampicillin resistance; Ery^R^, erythromycin resistance; Kan^R^, kanamycin resistance; Cm^R^, chloramphenicol resistance.

### Determination of the minimum inhibitory concentration of xanthorrhizol against *Staphylococcus aureus* strains

To measure the MIC values, a standard microdilution method was used according to a previously described procedure (Wiegand et al., 2008), with some modifications. Briefly, overnight cultures of the *S. aureus* strains grown in Mueller Hinton broth were reinoculated in the same fresh medium. When the cultures reached an OD_600_ of 0.5, the cells were adjusted to a final desired inoculation concentration of 5 × 10^5^ cfu/ml. The xanthorrhizol concentrations ranged from 1 to 1,024 μg/mL, prepared via serial twofold dilution. The MIC was determined as the lowest concentration of the antimicrobial compound at which the bacteria did not demonstrate visible growth. This experiment was carried out in duplicate.

### Optical diffraction tomography

To measure the 3D RI tomogram of individual bacterial cells, optical diffraction tomography (ODT) was performed using a Mach–Zehnder interferometer equipped with a digital micromirror device (Oh et al., 2020). *S. aureus* cells were cultivated in TSB medium to a final OD_600_ of 0.5 and prepared for ODT as previously described (Oh et al., 2020). To perform bacterial imaging, the *S. aureus* cells were resuspended in TSB medium and divided into two parts: one was supplemented with xanthorrhizol at a final concentration of 64 μg/mL, and the other was supplemented with DMSO. Ten microlitres of the resuspended solution was placed between two coverslips and then measured with an optical imaging system for 30 min at 5-min intervals. All the measurements were performed at room temperature (25°C). The 3D rendered image was acquired using commercial software (TomoStudioTM, Tomocube Inc., Daejeon, Republic of Korea), which more clearly defines the overall refractive index (RI) distribution of bacteria. Quantitative analysis of cell parameters was performed as previously described (Oh et al., 2020).

### Time-kill kinetics assay

Bacteria grown overnight in TSB medium were diluted 400-fold in fresh TSB medium, incubated at 37°C to a final OD_600_ of 0.5 and then diluted 10-fold. The cells were split into two groups and treated with xanthorrhizol (32–64 μg/mL) or with DMSO at a concentration equal to that used for drug-treated bacteria. The cells were then cultivated at 37°C for 1 or 5 h. Samples were taken after 37 s, 1.25, 2.5, 5, 10, and 30 min, and 1, 2 3, and 5 h; then, the samples were serially diluted 1:10 with sterile PBS containing 1 mM EDTA and 0.2% Kolliphor P188 (Sigma Aldrich, St. Louis, MO) and plated on solid TSA medium for colony counting. Kolliphor P188 was used to inactivate xanthorrhizol for live cell detection at each test point. The values represent the means of three independent experiments. The error bars indicate the standard errors.

### Molecular modeling for binding of xanthorrhizol to *Escherichia coli* MscL

The structure of EcMscL is not available in PDB. Therefore, the sequence of EcMscL was downloaded from UniProtKB. Structure of the *M*. *tuberculosis* MscL (PDB Code 2OAR) was used as the template for homology modeling (Wray et al., 2016). The homology model of EcMscL was built using Prime in Schrödinger Suite 2021-2 (Schrödinger, LLC, 2021a). Protein structures were prepared using the Protein Preparation Wizard. All missing loops were filled using Prime and implemented in Maestro version 2021-2 (Schrödinger, LLC, 2021b). Hydrogen atoms were added to the complex structure at pH 7.4, and their positions were optimized using PROPKA in Maestro. Restrained energy minimization was performed with an OPLS4 force field within 0.3 Å root mean square deviation (RMSD). Molecular docking was conducted to predict the binding poses of using the Glide (Maestro version 2021–2). Xanthorrhizol was docked with extra precision (XP) using Maestro’s Glide on the constructed homology model.

### Construction of the *Staphylococcus aureus* ATCC 29213Δ*mscL* mutant strain

In-frame deletion of the *mscL* gene was conducted using the pHoss1 suicide plasmid (Abdelhamed et al., 2015) and overlap extension PCR with the primers mL1 (5′-GATATCGGATCCATATGACGTCGACGTAGAGCACCCATACCTAACG-3′) and mL4 (5′-TTTCTCACGTAATAAATCCATTACACTCAACCTCTCTTT-3′) for amplification of the 1 kb upstream fragment and mL3 (5′-AAAGAGAGGTTGAGTGTAATGGATTTATTACGTGAGAAA-3′) and mL2 (5′-GTTTGAATCATTAGATCCCATGGGAAATGGAAATGTCGAGGCTG-3′) for amplification of the 1 kb downstream fragment of the *mscL* gene. Then, both PCR fragments were used as templates for the second PCR with the mL1 and mL2 primers, and the resulting 2 kb PCR fragment was inserted between the SalI/NcoI restriction sites of pHoss1 to generate the pHoss1/Δ*mscL* plasmid. The pHoss1/Δ*mscL* plasmid was subsequently transformed into the methylation-deficient strain *E. coli* ET12567. Demethylated plasmid pHoss1/Δ*mscL* DNA was delivered into the *S. aureus* ATCC 29213 cells by electroporation. Electrocompetent *S. aureus* cells were prepared and electroporated as previously described (Schneewind and Missiakas, 2014). The transformed cells were recovered in TSB medium at 30°C for 3 h and then incubated on TSA solid medium supplemented with erythromycin (20 μg/mL) for 48 h at 30°C. One erythromycin-resistant clone was selected and incubated for 24 h in TSB medium supplemented with erythromycin (20 μg/mL) at 42°C. Then, 1 μl of the cell culture was diluted 1,000-fold with fresh TSB (without Ery) and incubated for 24 h at 30°C, which was repeated twice. Thereafter, the culture was reinoculated into fresh TSB, incubated for 8 h at 42°C, serially diluted and plated onto solid TSA supplemented with anhydrotetracycline (2.5 μg/mL). After 2 days of cultivation at 30°C, clones sensitive to erythromycin were selected and checked for loss of the *mscL* gene by PCR.

### Construction of the pBEP-SaMscL expression plasmid

The *B. subtilis*/*E. coli* shuttle vector pBE-S (Takara Bio, Inc., Shiga, Japan) with an origin of replication derived from pUB110 was used to construct a protein expression vector suitable for *S. aureus.* The SpeI/HindIII fragment of pBE-S was replaced with the NheI/HindIII fragment from the plasmid pHT01 carrying the *lacI* gene and the IPTG-inducible Pgrac promoter to generate the pBEP expression vector. The *mscL* gene was amplified from the genomic DNA of *S. aureus* ATCC 29213 using the primers mL5 (5′-CAATTAAAGGAGGAAGGATCCATGTTAAAAGAATTCAAAGAG-3′) and mL6 (5′-GATGTCTAGACTGCAGGTCGACTTTTTTCTCACGTAATAAATC-3′) and inserted into the BamHI/SalI sites of the pBEP plasmid in frame with a C-terminal 6x-histidine tag to construct pBEP-SaMscL (Supplementary Figure S1). The pBEP and pBEP-SaMscL plasmids were transferred to the *S. aureus* ATCC 29213Δ*mscL* mutant strain, and IPTG-induced SaMscL protein expression was confirmed by SDS–PAGE and Western blotting using Histidine (C-term) tag (6XHis) -AP Mouse Monoclonal Antibody (Thermo Fisher Scientific, Waltham, MA, United States) (Supplementary Figure S1). Site-directed mutagenesis of the *mscL* gene to introduce the substitutions E4C, F5C, and F8C was performed using the forward primers SauC4, SauC5 and SauC8 listed in Supplementary Table S1 and the reverse primer mL6. The SaMscL mutants V14C, M23C, F73C, A77C, F78C, A79C, V84C, and F85C were constructed through overlap extension PCR using a QuickChange II-E Site-Directed Mutagenesis Kit (Agilent, Santa Clara, CA, United States) with the primer pairs shown in Supplementary Table S1. Changes in the sequence are underlined.

### MIC curves for xanthorrhizol against exponentially growing staphylococcal cultures

Overnight cultures of wild-type *S. aureus* ATCC 29213 and its *mscL* null mutant grown in TSB medium were diluted 400-fold in the same medium and incubated with vigorous shaking for 1.5 h at 37°C. Thereafter, 1 μL of cell suspension was added to 99 μL of xanthorrhizol-enriched TSB medium prepared by twofold dilution in a 96-well plate in 3 replicates. The plates were incubated in an Infinite 200 Pro microplate reader (Tecan Austria GmbH, Grödig, Austria) at 37°C for 16 h. The OD_600_ was measured every 10 min.

The MIC curves for xanthorrhizol against the exponentially growing *S. aureus* ATCC 29213Δ*mscL* mutant strain with controlled expression of the SaMscL protein were obtained with some modifications. Overnight cultures of *S. aureus* ATCC 29213Δ*mscL* harboring the pBEP and pBEP-SaMscL plasmids grown in TSBKan_25_ medium were diluted 100-fold in fresh medium and incubated for 30 min at 37°C. Expression was then induced with 1 mM IPTG, and the cells were grown for 1 h. One microlitre of cell suspension was added to 99 μL of xanthorrhizol-enriched TSBKan_25_IPTG_1mM_ medium prepared by twofold dilution in a 96-well plate in 3 replicates and incubated in an Infinite 200 Pro microplate reader (Tecan Austria GmbH, Grödig, Austria) at 37°C for 16 h. The OD_600_ was measured every 10 min.

### Viability of staphylococcal cultures overexpressing the SaMscL protein in the exponential and stationary growth phases

The viability of exponentially growing *S. aureus* ATCC 29213Δ*mscL* cultures harboring the empty vector plasmid pBEP or pBEP-SaMscL was studied 1 h after IPTG induction. Overnight cultures grown in TSBKan_25_ medium were diluted 1/100 in the same medium, cultivated for 30 min under vigorous aeration at 37°C and induced for 1 h with 1 mM IPTG. After this, the cells were washed with PBS, diluted in PBS to a final concentration of approximately 10^6^ CFU/mL, and treated with 12.8 μg/mL xanthorrhizol. Samples were collected at 5, 10, 20, and 40 min and 1 h, serially diluted 1:10 with sterile PBS containing 1 mM EDTA and 0.2% Kolliphor P188 (Sigma Aldrich, St. Louis, MO) and plated on solid TSA medium for colony counting.

Overnight cultures of *S. aureus* ATCC 29213 and its *mscL* null mutant grown in TSB were treated with 64 μg/mL xanthorrhizol for 6 h at 37°C. Sterile DMSO was used in the control experiment. Afterwards, the cultures were diluted 20-fold (Wray et al., 2020) with sterile PBS containing 1 mM EDTA and 0.2% Kolliphor P188 (Sigma Aldrich St. Louis, MO, United States), serially diluted 1:10 in the same buffer and plated on solid TSA medium for colony counting.

*S. aureus* ATCC 29213Δ*mscL* cells harboring the pBEP and pBEP-SaMscL plasmids were induced with 1 mM IPTG at an OD_600_ of 0.3 and incubated overnight at 37°C. Overnight cultures were divided into two parts: one was treated with 64 μg/mL xanthorrhizol, and sterile DMSO was added to the other. After 6 h of incubation at 37°C, samples were taken and processed as described above.

### Glutamate and potassium assays in stationary-phase cultures

Sample preparation for the glutamate assay was performed as previously described (Wray et al., 2020), with some modifications. Briefly, overnight cultures of the *S. aureus* ATCC 29213Δ*mscL* mutant strain carrying the plasmids pBEP and pBEP-SaMscL were diluted 100-fold in fresh TSBKan_25_ medium, incubated at 37°C and induced with 1 mM IPTG for 1 h after reaching an OD_600_ of 0.25. Afterwards, each culture was divided into two parts: one was treated with 25 μg/mL xanthorrhizol, and sterile DMSO was added to the other. The culture was incubated for 17 h at 37°C, after which the final OD_600_ was recorded. For each sample, 6 mL of overnight culture was pelleted, and then the pellets were resuspended in TSB medium, adjusting the volume for the final OD_600_, and sonicated for 2 min. Glutamate measurements were performed using the EnzyChrom Glutamate Assay Kit (BioAssay Systems, Hayward, CA, United States) in a 96-well plate with 3 replicates, and the OD at 565 nm was read in an Infinite 200 Pro microplate reader (Tecan Austria GmbH, Grödig, Austria) at 25°C. The amount of intracellular K+ was measured in the same samples using the Potassium (K) Turbidimetric Assay Kit (MyBioSource, San Diego, CA, United States) as described in the manual in a 96-well plate; 3 replicates of the OD at 450 nm were read in an Infinite 200 Pro microplate reader (Tecan Austria GmbH, Grödig, Austria) at 25°C. Viability assays were performed simultaneously.

### Statistical analyses

All experiments were performed in triplicate, and the mean ± SD values are displayed in each graph. The significance of differences between mean values of two measured parameters was assessed using a 2-tailed, paired Student’s *t* test with unequal variances. Differences were considered significant when the *p* value was < 0.05.

## Results

### Rapid killing effect of xanthorrhizol against MRSA and MSSA strains

**Figure 1:**
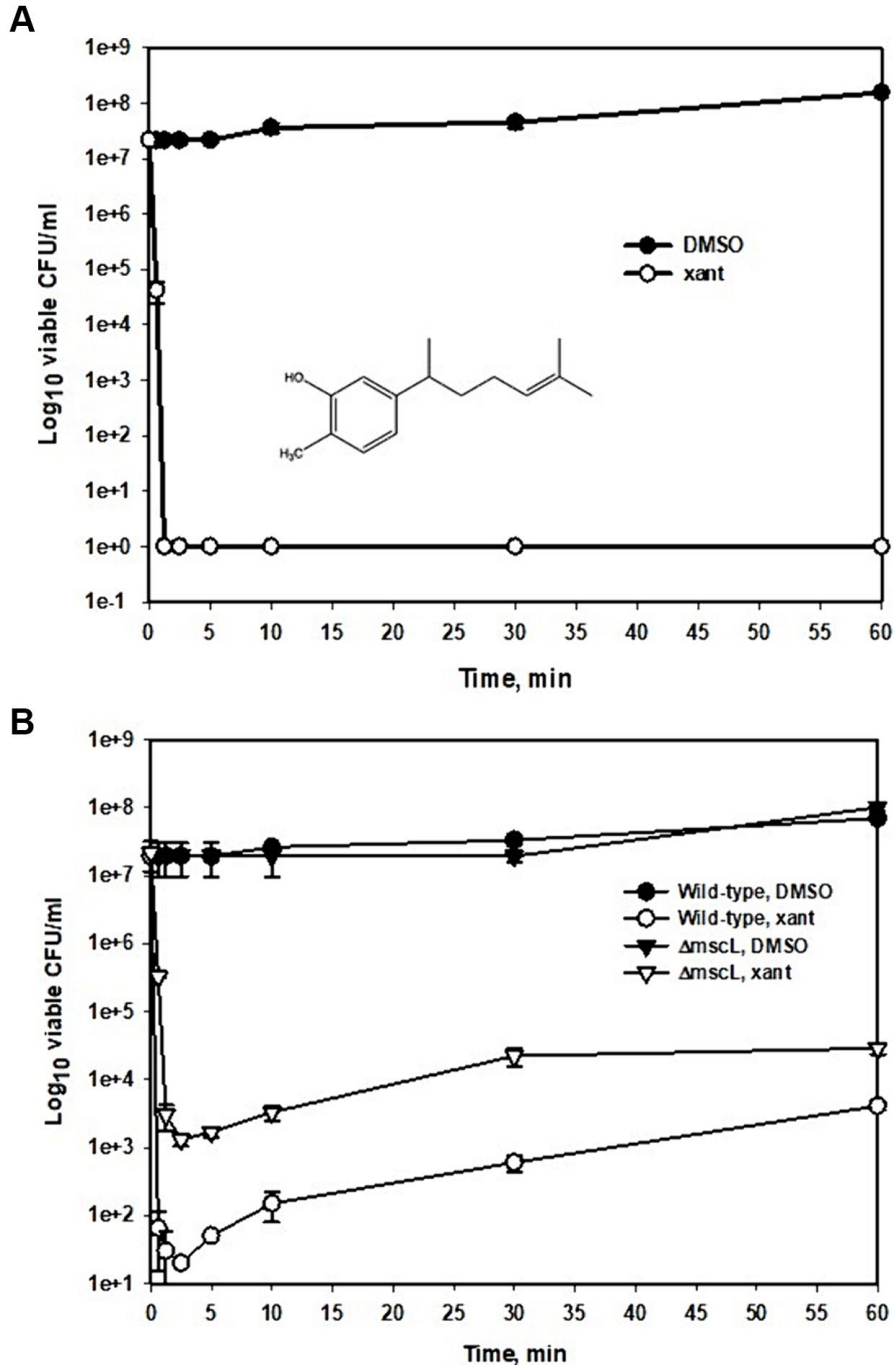
Time-kill kinetic curves of xanthorrhizol against MRSA ATCC 33592 or against MSSA ATCC 29213 and ATCC 29213Δ*mscL*. Exponentially growing cultures of ATCC 33592 **(A)** or ATCC 29213 and its *mscL* null mutant **(B)** were exposed to 64 μg/mL **(A)** and 32 μg/mL **(B)** xanthorrhizol and incubated at 37°C for 1 h. Error bars represent standard deviations from three independent experiments.

The MIC of xanthorrhizol was determined to be 64 μg/mL for both the MRSA strain ATCC 33592 and the MSSA strain ATCC 29213. Xanthorrhizol at this concentration (1xMIC) was used for the time-kill assay against ATCC 33592 cells (Figure 1A). Samples taken at 1 min and 15 s and later after xanthorrhizol treatment showed no living cells (Figure 1A). We also found no living cells 5 h after exposure to 1 × MIC xanthorrhizol (data not shown).

Since the MRSA strain ATCC 33592 is resistant to many antibiotics used in genetic experiments, such as erythromycin (100 μg/mL), kanamycin (25 μg/mL), and tetracycline (30 μg/mL) (our unpublished data), we plotted a time–kill curve for the MSSA strain ATCC 29213 (Figure 1B) using 0.5 × MIC xanthorrhizol for a more detailed study of its action against *S. aureus.* This strain also exhibited high sensitivity to xanthorrhizol when the number of living cells decreased from 2 × 10^7^ CFU/ml to 65 CFU/mL during the first 37 s of incubation with xanthorrhizol and remained at the same level until 5 min after treatment (Figure 1B). From the 10th minute, the number of living cells in the experimental group began to increase, possibly because the time-kill assay was carried out under conditions conducive to growth, and from the 30th minute, the difference between the number of living cells treated with xanthorrhizol and those not treated decreased from 5 and a half orders of magnitude to 4 after 1 h of incubation, amounting to approximately 4 × 10^3^ CFU/ml (Figure 1B).

### The effect of xanthorrhizol on *Staphylococcus aureus* cell morphology appears to be MscL dependent

**Figure 2:**
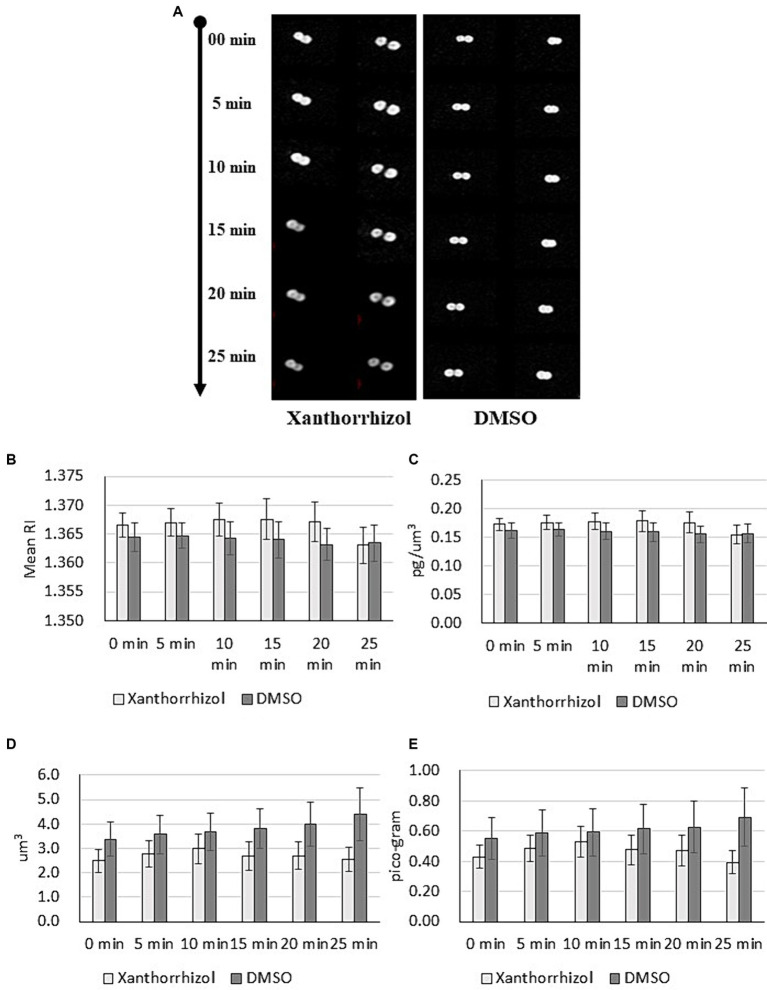
Maximum RI projection images **(A)** and quantitative analysis results **(B–E)** for *S. aureus* ATCC 29213 cells treated with xanthorrhizol or DMSO over time. The mean RI **(B)**, cytoplasm concentration **(C)**, cell volume **(D)** and cellular dry mass **(E)** for the control (*n* = 11) and xanthorrhizol (*n* = 12) groups were determined over time. The error bars indicate the standard errors.

To investigate changes in individual cells of *S. aureus* ATCC 29213 treated with xanthorrhizol, we measured 3D RI tomograms of bacteria using optical diffraction tomography (ODT) (Oh et al., 2020) and analyzed the resulting images. We visualized the dynamics of *S. aureus* cells in growth medium supplemented with or without xanthorrhizol (1xMIC) for 5 min (Figure 2A). As shown in Figure 2A, DMSO-treated control cells appeared healthy during the observation period. In contrast, *S. aureus* cells treated with xanthorrhizol over time began to look like “shadows” when cytoplasmic components were released, resulting in empty cell membranes (Figure 2A). In addition, between 20 and 25 min of exposure to xanthorrhizol, the RI value suddenly decreased (Figure 2B; Supplementary Table S2), which could be the time point at which bacterial lysis was initiated, since a decrease in the RI value of bacteria indicates the efflux of cell cytoplasm (Oh et al., 2020). This result is consistent with the cytoplasmic concentration data (Figure 2C; Supplementary Table S2), which showed a rapid decrease between 20 and 25 min of observation, which means that after bacterial lysis, the internal contents were released outside the cell wall, resulting in a decrease in the cytoplasmic concentration (Oh et al., 2020). Other morphological characteristics of bacterial growth under xanthorrhizol treatment, such as cell volume and cellular dry mass, also demonstrated a reduction in values starting from the 15th minute (Figures 2D,E; Supplementary Table S2). Moreover, the formation of “shadow cells,” as shown in Figure 2A, was found to be consistent over time, with a decrease in cell volume and dry mass for xanthorrhizol-treated cells. In DMSO-treated control *S. aureus* cells, the RI and cytoplasm concentration remained unchanged during the observation period (Figures 2B,C; Supplementary Table S2), and the cell volume and cellular dry mass increased (Figures 2D,E; Supplementary Table S3), indicating continuous growth.

Thus, the morphological changes found in xanthorrhizol-treated *S. aureus* cells are consistent with the spontaneous activation of MscL, resulting in the loss of solutes and osmolytes and hence a decrease in the size of *S. aureus*.

### Involvement of the MscL channel in xanthorrhizol-induced inhibition of *Staphylococcus aureus* growth

To determine whether the xanthorrhizol susceptibility of *S. aureus* was dependent on the MscL mechanosensitive channel, we constructed a *S. aureus* ATCC 29213 mutant lacking the *mscL* gene and compared the results of a time-kill assay of this mutant with those of the wild-type strain (Figure 1B). The number of living cells of the *mscL* null mutant after 37 s of exposure to xanthorrhizol was 2 orders of magnitude greater than that of the wild-type strain. The difference in live cell numbers between the wild type and the Δ*mscL* mutant then began to decrease, reaching approximately 1 order of magnitude after 1 h of xanthorrhizol treatment (Figure 1B).

**Figure 3:**
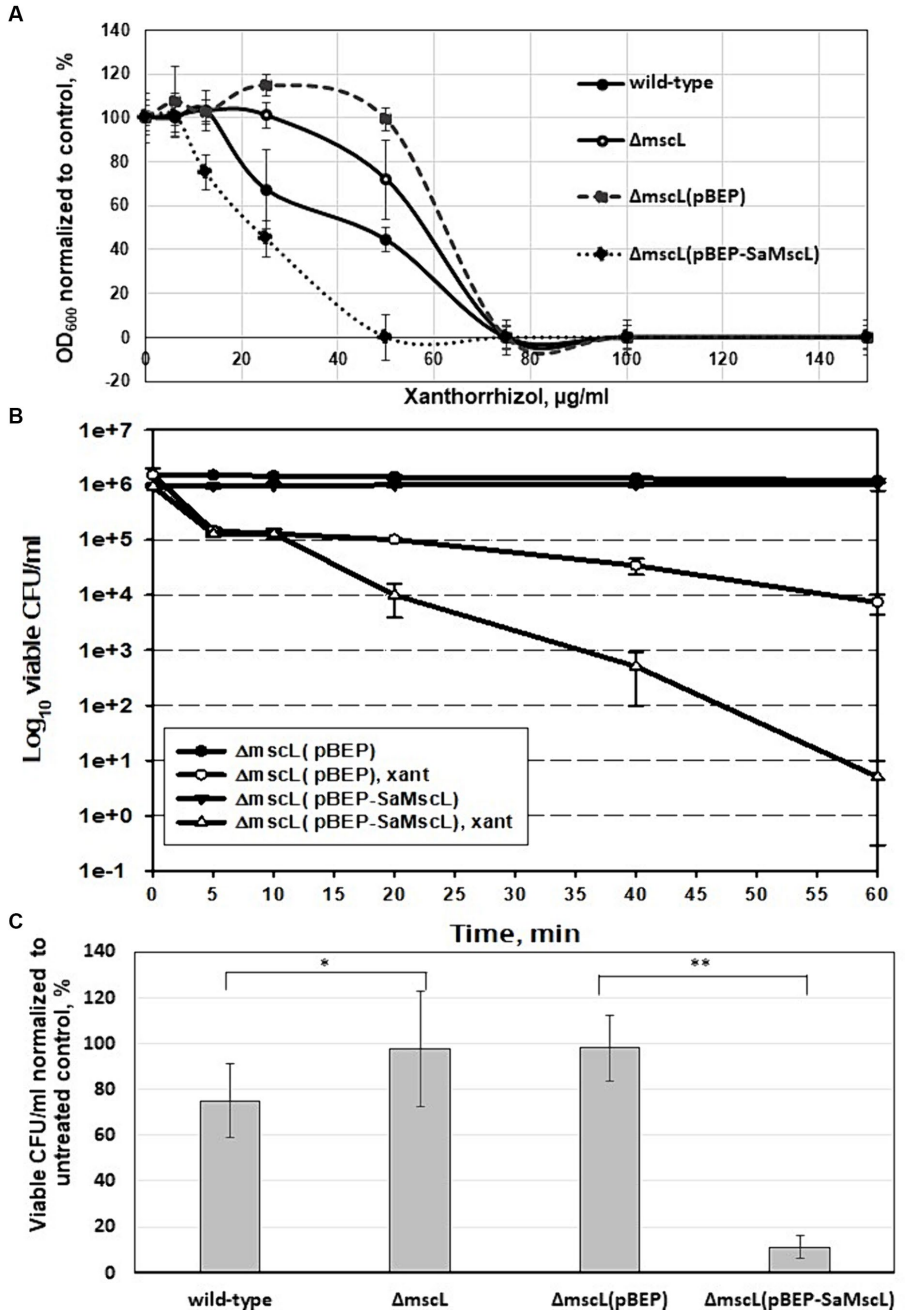
Effect of SaMscL protein deficiency or overproduction on the viability of *S. aureus* ATCC 29213 and *S. aureus* ATCC 29213Δ*mscL* in the presence of xanthorrhizol. Growth inhibition by increasing concentrations of xanthorrhizol was shown for exponentially growing *S. aureus* ATCC 29213, *S. aureus* ATCC 29213Δ*mscL*, *S. aureus* ATCC 29213Δ*mscL* (pBEP) and *S. aureus* ATCC 29213Δ*mscL* (pBEP-SaMscL) cultures **(A)**. The decrease in growth is reflected as the percentage of the OD_600_ of the treated cultures to that of the untreated control. The error bars indicate the standard errors. Time-kill kinetic curves of xanthorrhizol against exponentially growing *S. aureus* ATCC 29213Δ*mscL* cells carrying the plasmids pBEP or pBEP-SaMscL were obtained 1 h after IPTG induction **(B)**, as described in the Materials and Methods section. The viability of stationary-phase cultures of *S. aureus* ATCC 29213, *S. aureus* ATCC 29213Δ*mscL, S. aureus* ATCC 29213Δ*mscL* (pBEP) and *S. aureus* ATCC 29213Δ*mscL* (pBEP-SaMscL) was studied after treatment with xanthorrhizol for 6 h **(C)**, where *p < 0.01 (*n* = 3); **p < 0.002 (*n* = 6) by a 2-tailed, paired Student’s *t* test.

We tested the effect of xanthorrhizol on the growth of *S. aureus* ATCC 29213 cells expressing the SaMscL channel at the endogenous level or under IPTG induction in dose dependence experiments (Figure 3A). The naturally expressed protein SaMscL appears to be involved in the xanthorrhizol-induced growth inhibition of wild-type *S. aureus* ATCC 29213 since the OD_600_ values of overnight cultures of this strain grown in the presence of 25 and 50 μg/mL xanthorrhizol were 34 and 28% lower, respectively than those of the Δ*mscL* mutant (Figure 3A). This effect was enhanced when the SaMscL protein was overexpressed in the *S. aureus* ATCC 29213Δ*mscL* mutant strain (Figure 3A). The OD_600_ values of IPTG-induced *S. aureus* ATCC 29213Δ*mscL* (pBEP-SaMscL) cells were 2.5- and 100-fold lower than those of cells carrying the empty vector in the presence of 25 μg/mL and 50 μg/mL xanthorrhizol (Figure 3A).

Additionally, accelerated cell death was demonstrated in exponentially growing *S. aureus* ATCC 29213Δ*mscL* cells overexpressing the SaMscL protein compared to empty vector-containing cells treated with 12.8 μg/mL xanthorrhizol (1/5 MIC) (Figure 3B). Therefore, after 1 h of incubation with xanthorrhizol, the number of living *S. aureus* ATCC 29213Δ*mscL* (pBEP-SaMscL) cells was found to be 3 orders of magnitude lower than that of *S. aureus* ATCC 29213Δ*mscL* (pBEP) cells (Figure 3B).

Previous studies have shown that some antimicrobial agents can significantly reduce the viability of “dormant”/stationary-phase *S. aureus* cells in a MscL-dependent manner (Wray et al., 2019a, 2020). We tested the viability of *S. aureus* ATCC 29213 and its Δ*mscL* mutant cells that reached the stationary phase and were then exposed to 1xMIC xanthorrhizol for 6 h. As shown in Figure 3C, the Δ*mscL* mutant was more resistant to xanthorrhizol and exhibited close to 100% survival, in contrast to wild-type cells, the viability of which was only 75%. Overexpression of SaMscL in *S. aureus* ATCC 29213Δ*mscL* (pBEP-SaMscL) resulted in a sharp decrease in viability (Figure 3C), with only 11% of the cells surviving after xanthorrhizol treatment, in contrast to cells carrying the empty plasmid, 98% of which survived. Thus, the ability of xanthorrhizol to kill stationary-phase *S. aureus* cells in a MscL-dependent manner may have important medical significance, since *S. aureus* biofilms found in infection sites (Moormeier and Bayles, 2017) are difficult to treat. Xanthorrhizol has previously been shown to have strong activity against *S. mutans* biofilms (Rukayadi and Hwang, 2006), possibly due to its action against MscL.

### Xanthorrhizol increases the potency of dihydrostreptomycin, known for its MscL channel targeting ability

**Figure 4:**
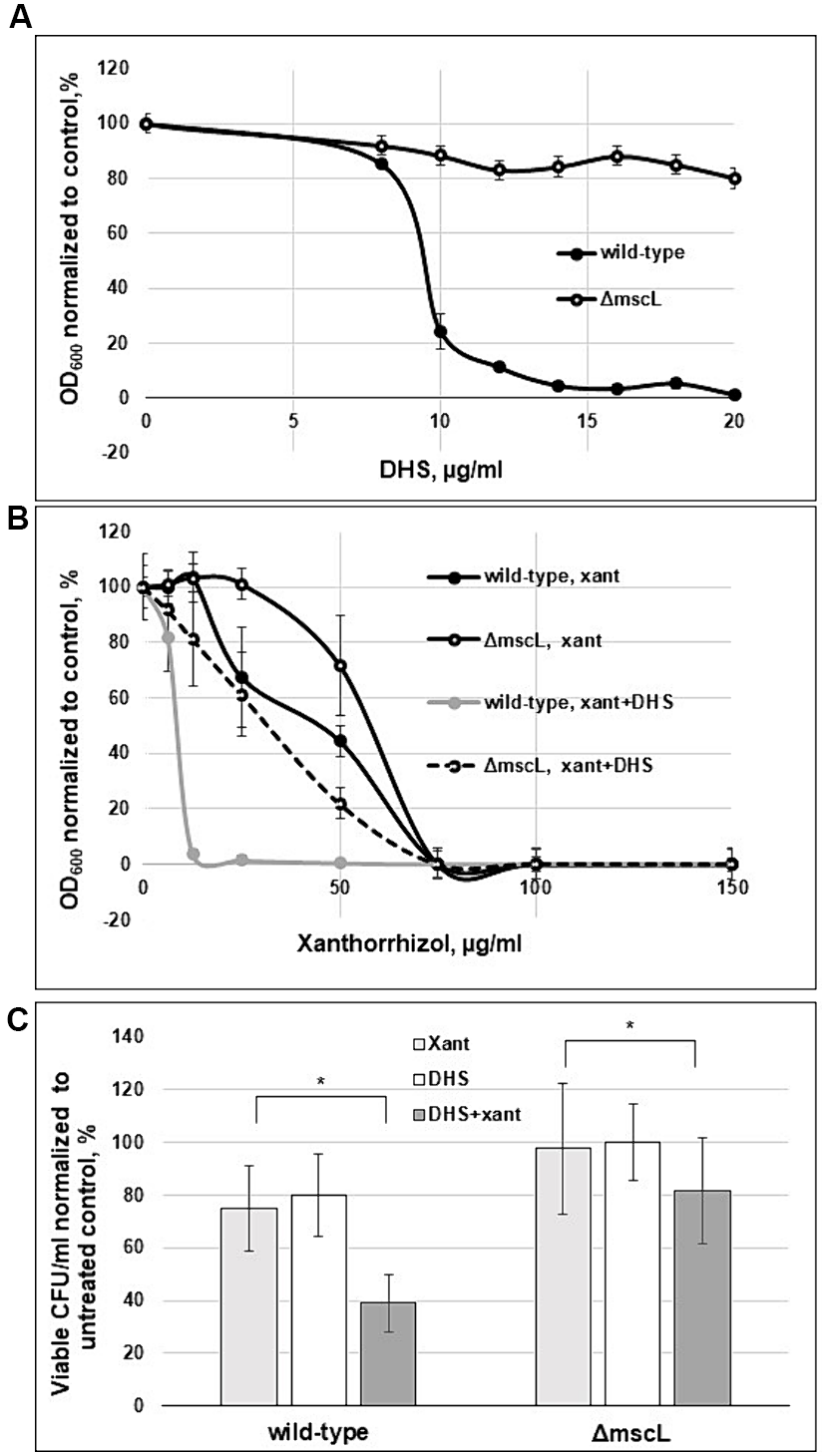
Xanthorrhizol increases the potency of DHS in a MscL-dependent manner. The concentration-dependent effects of DHS **(A)** and xanthorrhizol in the presence of DHS (8 μg/mL) are shown **(B)** for exponentially growing *S. aureus* ATCC 29213 and *S. aureus* ATCC 29213Δ*mscL* cells. The decrease in growth is reflected as the percentage of the OD_600_ of the treated cultures to that of the untreated control. The error bars indicate the standard errors. The viability of stationary-phase cultures of *S. aureus* ATCC 29213 and *S. aureus* ATCC 29213Δ*mscL* **(C)** was studied after treatment with 50 μg/mL DHS or a combination of DHS (50 μg/mL) and xanthorrhizol (64 μg/mL) for 6 h, where *p < 0.01 (*n* = 3); *p < 0.05 (*n* = 3) by a 2-tailed, paired Student’s *t* test.

To provide further evidence of the effectiveness of xanthorrhizol against the SaMscL channel, we tested whether xanthorrhizol could increase the potency of dihydrostreptomycin (DHS), a cidal antibiotic, in targeting *E. coli* MscL and its orthologues from *B. subtilis* and *S. aureus* expressed in the *E. coli* Δ*mscL* mutant (Iscla et al., 2014; Wray et al., 2016). The MIC of DHS was determined to be 16 μg/mL for wild-type *S. aureus* ATCC 29213 and 32 μg/mL for *S. aureus* ATCC 29213Δ*mscL.* To obtain more detailed information on the activity of DHS against *S. aureus*, we studied the growth inhibition of exponentially growing cells of the ATCC 29213 strain and its Δ*mscL* mutant in a dose dependence experiment. As shown in Figure 4A, the OD_600_ values of the wild-type and Δ*mscL* overnight cultures were 85 and 91%, respectively, compared to those of the untreated cells at a DHS concentration of 8 μg/mL. However, starting at a DHS concentration of 10 μg/mL, the wild-type OD_600_ decreased sharply to 24% and subsequently reached 1% at 20 μg/mL DHS, in contrast to the Δ*mscL* mutant, which retained the same level of viability at DHS concentrations ranging from 10 to 20 μg/mL (Figure 4A). The results of this experiment clearly confirmed that DHS targets SaMscL.

We then tested whether xanthorrhizol could increase the potency of the subthreshold concentration of DHS. Exponentially growing cells of the ATCC 29213 strain and its Δ*mscL* mutant were cultivated in the presence of 8 μg/mL DHS and various concentrations of xanthorrhizol (Figure 4B). After combined treatment with DHS and xanthorrhizol, the growth of the wild-type strain greatly decreased compared with that after treatment with DHS or xanthorrhizol alone (Figures 4A,B). Compared with the wild-type strain, the Δ*mscL* mutant was more resistant to the simultaneous action of DHS and xanthorrhizol (Figure 4B). However, its viability was also lower with the combination of DHS and xanthorrhizol than with each antimicrobial agent used separately (Figures 4A,B).

We found a similar effect after combinatorial treatment of stationary-phase *S. aureus* ATCC 29213 and *S. aureus* ATCC 29213Δ*mscL* cells, wherein the viability of both strains decreased by approximately half or 11% for the wild-type strain or the Δ*mscL* mutant, respectively, compared to that in the separate treatment with DHS or xanthorrhizol (Figure 4C). Interestingly, the stationary-phase *S. aureus* ATCC 29213 cells were more resistant to DHS, and to observe a decrease in viability, we increased the concentration of DHS from 16 μg/mL (1 × MIC) to 50 μg/mL. In this experiment, the Δ*mscL* mutant also exhibited increased resistance to both antimicrobials in combination or separately (Figure 4C).

The data obtained here showed that xanthorrhizol increases the likelihood of DHS passing through the SaMscL channel, followed by a decrease in the MIC for DHS. However, a visible decrease in the viability of mutant Δ*mscL* cells after combined treatment with DHS and xanthorrhizol may indicate that SaMscL is not a unique pathway for entry into *S. aureus* cells.

### Xanthorrhizol induces potassium (K^+^) and glutamate efflux from *Staphylococcus aureus* ATCC 29213*ΔmscL* (pBEP-SaMscL) cells

**Figure 5:**
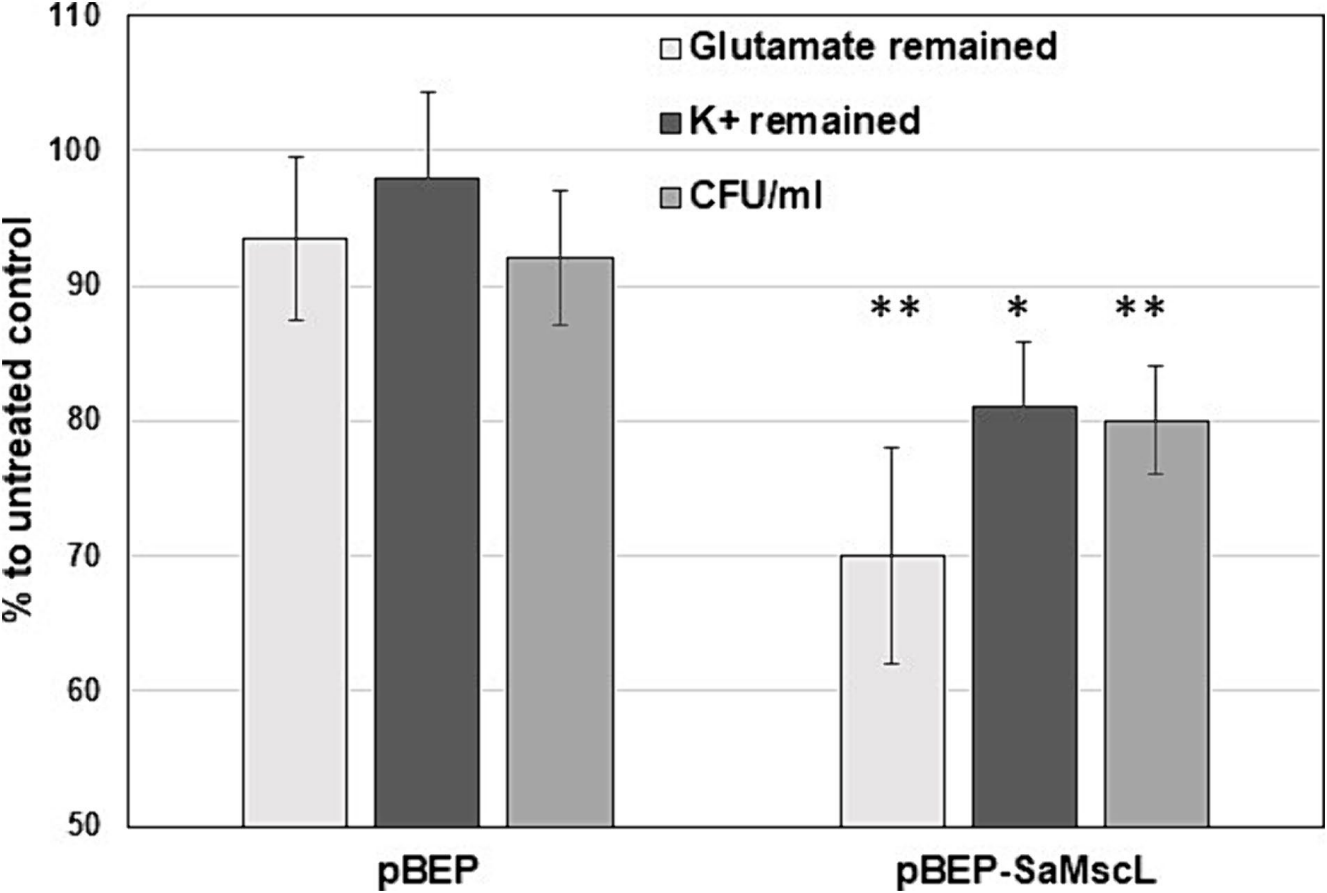
The decrease in the steady-state levels of glutamate and K^+^ in *S. aureus* ATCC 29213Δ*mscL* cells treated with xanthorrhizol is associated with the SaMscL protein The glutamate and K^+^ levels in *S. aureus* ATCC 29213Δ*mscL* cells harboring the empty expression vector pBEP or pBEP-SaMscL after overnight incubation with xanthorrhizol are shown as percentages of those in the untreated controls. Error bars reflect standard deviations from three independent measurements for glutamate, **p < 0.002; for K^+^, *p < 0.01 by a 2-tailed, paired Student’s *t* test. The viability of the bacterial cells was measured at the same time and is displayed as a percentage of the viability of the untreated controls. Error bars represent standard deviations from three independent measurements, **p < 0.002 by a 2-tailed, paired Student’s *t* test.

Previous studies have shown that treatment of *E. coli* cells overexpressing the McsL channel with some antimicrobial agents increased the efflux of K^+^ and glutamate from the cytoplasm due to inappropriate gating of MscL (Iscla et al., 2014; Wray et al., 2020). We tested the K^+^ and glutamate levels in IPTG-induced *S. aureus* ATCC 29213*ΔmscL* (pBEP-SaMscL) cells treated with xanthorrhizol. As shown in Figure 5, the relative amount of K^+^ decreased by 19% and that of glutamate decreased by 30% after treatment with xanthorrhizol compared to 2 and 6%, respectively, in *S. aureus* ATCC 29213Δ*mscL* cells carrying the empty vector. Thus, the flux data obtained are consistent with the idea that SaMscL is gated *in vivo* in response to xanthorrhizol treatment.

### Identification of amino acid residues contributing to the binding of xanthorrhizol to the SaMscL channel

**Figure 6:**
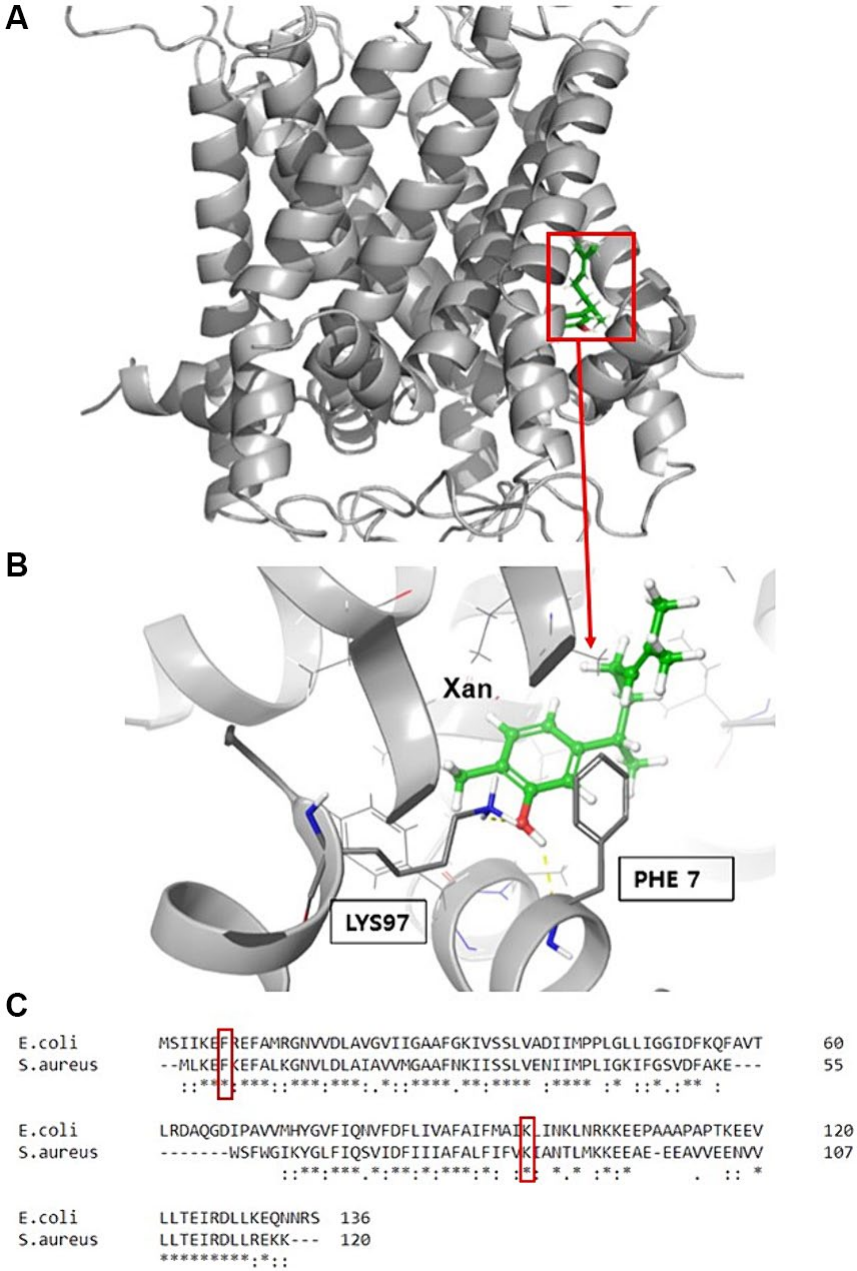
Molecular modeling of EcMscL with the docking site for xanthorrhizol. Xanthorrhizol binding pocket **(A)** in the EcMscL complex and an enlarged view of xanthorrhizol relative to the positions of residues F7 and K97 **(B)**. Sequence alignment of the MscLs from *E. coli* and *S. aureus* with the corresponding residues **(C)**.

Previously, successful attempts to determine the binding sites for some antibacterial agents in MscL by comparison to the *E. coli* channel (EcMscL) have been described using a library of cysteine mutants (Wray et al., 2016, 2019b, 2020). Based on molecular modeling of EcMscL (Wray et al., 2019b), we identified two amino acid residues, F7 and K97, that possibly contribute to the binding of xanthorrhizol (Figures 6A,B). Sequence alignment between the MscL orthologues from *E. coli* and *S. aureus* showed that F5 and K85 in SaMscL are analogs of F7 and K97 from EcMscL (Figure 6C). To determine other amino acids involved in xanthorrhizol binding, we checked several residues from SaMscL (E4, F5, F8, V14, M23, F73, A77, F78, A79, V84, and K85). The reason for choosing the amino acid residues mentioned above was that their counterparts in EcMscL have been shown or suspected to be involved in binding to other antimicrobials (Wray et al., 2019b, 2020, 2022). Single substitutions of the aforementioned amino acid residues for cysteine were introduced into SaMscL, a cysteine-free protein, and the expression plasmids pBEP-SaMscL carrying each mutated *mscL* gene were transformed into the *S. aureus* ATCC 29213Δ*mscL* strain. Exponential- and stationary-phase *S. aureus* ATCC 29213Δ*mscL* (pBEP-SaMsc) cells overexpressing cysteine mutants of SaMscL were treated with xanthorrhizol at 1/5 × MIC or 1 × MIC, respectively, as described in the Materials and Methods section.

**Figure 7:**
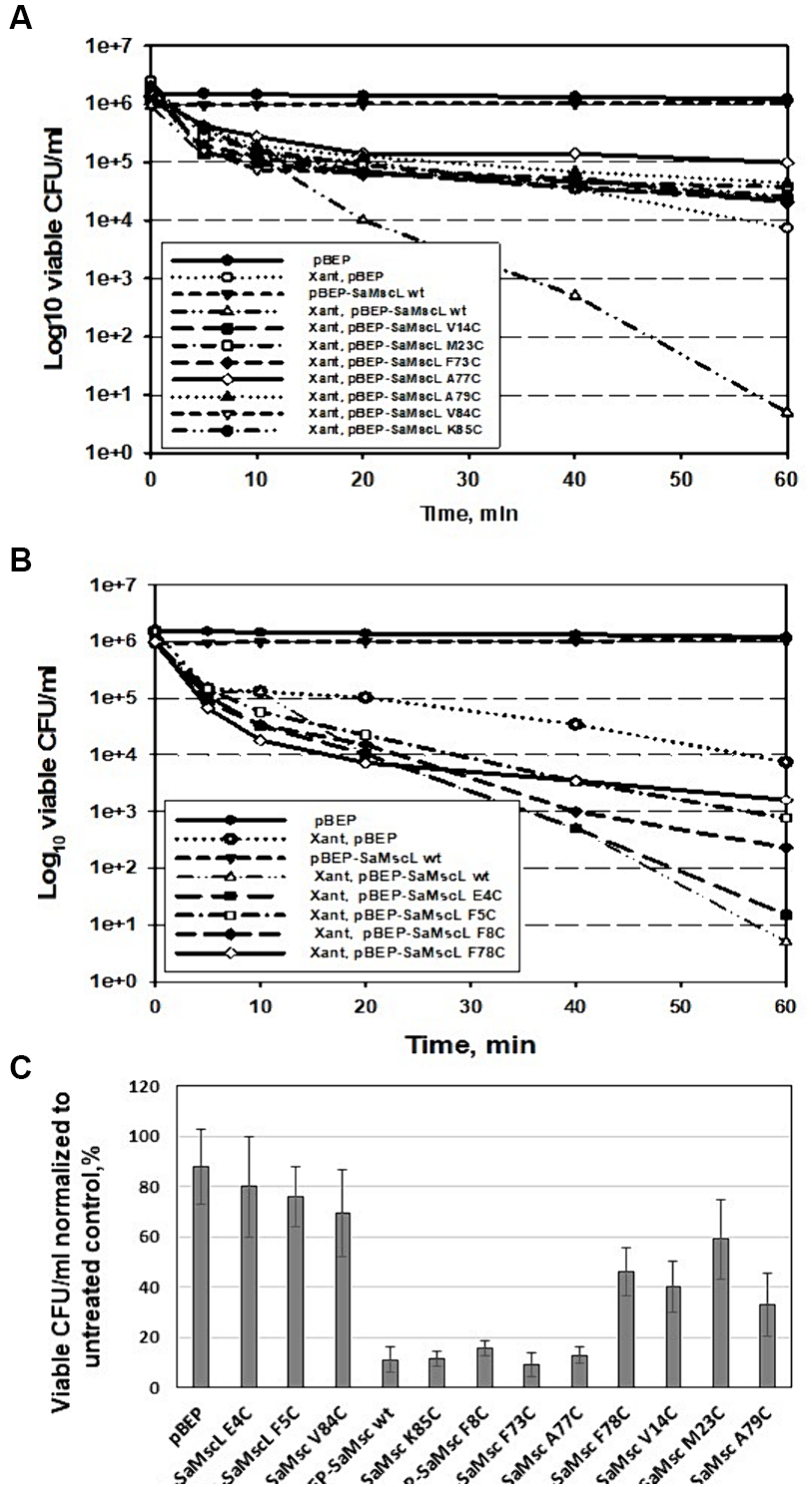
Detection of key amino acid residues involved in the interaction of SaMscL with xanthorrhizol. Time-kill kinetic curves of xanthorrhizol against exponentially growing *S. aureus* ATCC 29213Δ*mscL* cells carrying a plasmid (pBEP) with or without mutated *mscL* genes **(A,B)**. The viability of stationary-phase cultures of *S. aureus* ATCC 29213Δ*mscL* carrying the empty vector or expressing wild-type and mutants of SaMscL was studied after treatment with 64 μg/mL xanthorrhizol for 6 h **(C)**.

Based on the viability data for exponentially growing cells, the SaMscL cysteine mutants could be divided into three groups. Group 1 included the V14C, M23C, F73C, A77C, A79C, V84C, and K85C mutants, which showed almost the same viability in the presence of xanthorrhizol as cells carrying the empty vector (Table 2; Figure 7A). The mutant from group 2, E4C, was as sensitive to xanthorrhizol as cells carrying wild-type SaMscL (Table 2; Figure 7B). Group 3, consisting of mutants F5C, F8C and F78C, demonstrated moderate tolerance/sensitivity to xanthorrhizol, with a decrease in the number of living cells after 1 h of incubation with xanthorrhizol of 3–4 orders of magnitude, compared to 1–2 orders of magnitude in group 1 and 5 orders of magnitude in group 2 (Table 2; Figure 7B).

**Table 2:** Comparative analysis of single amino acid substitutions in the SaMscL protein affecting its tolerance to xanthorrhizol.

*S. aureus* ATCC 29213Δ*mscL* harboring	Cell viability in the exponential phase of growth^a^	Cell viability in the stationary phase of growth^b^
pBEP (vector)	+++	+++
pBEP-SaMscL wt	+	+
pBEP-SaMscL E4C	+	+++
pBEP-SaMscL F5C	++	+++
pBEP-SaMscL F8C	++	+
pBEP-SaMscL V14C	+++	++
pBEP-SaMscL M23C	+++	++
pBEP-SaMscL F73C	+++	+
pBEP-SaMscL A77C	+++	+
pBEP-SaMscL F78C	++	++
pBEP-SaMscL A79C	+++	++
pBEP-SaMscL V84C	+++	+++
pBEP-SaMscL K85C	+++	+

a Decrease in the number of living cells in the exponential growth phase by 1–2 orders of magnitude +++; 3–4 orders of magnitude ++; 5 orders of magnitude +.

b Decrease in the number of living cells in the stationary phase by 20% (+++); 40–60% ++; >80% +.

The viability of stationary-phase cells was found to be different from that of exponentially growing cells. Therefore, group 1 consisted of the E4C, F5C, and V84C mutants, whose tolerance to xanthorrhizol was almost the same as that of cells carrying the empty vector (78, 80, and 69.5%, respectively) (Table 2; Figure 7C). The composition of group 2 was changed to include the mutants F8C, F73C, A77C, and K85C, which exhibited xanthorrhizol sensitivity similar to that of cells carrying wild-type SaMscL (Table 2; Figure 7C). Group 3, with moderate tolerance/sensitivity to xanthorrhizol in the stationary phase, consisted of the mutants V14C, M23V, F78C, and A79C, with cell viabilities of 33–59% (Table 2; Figure 7C).

By comparing viability data from experiments using exponential- and stationary-phase *S. aureus* cells, the amino acid residues F5, V14, M23, A79, and V84 were predicted to be the residues for the binding of xanthorrhizol.

## Discussion

In the present paper, we report that xanthorrhizol is active against MRSA and MSSA strains at therapeutic concentrations (Figure 1). The undoubted advantage of xanthorrhizol (1 × MIC) is its rapid killing effect on MRSA, which prevents the formation of resistant clones. The 3D RI tomogram of individual staphylococcal cells showed morphological changes after treatment with xanthorrhizol, which are consistent with spontaneous activation of SaMscL, such as a reduction in size and the emergence of “shadow cells” as a result of the loss of solutes and osmolytes (Figure 2). Here, we showed that the efficacy of xanthorrhizol against *S. aureus* was, at least partially, associated with the mechanosensitive channel SaMscL. The results obtained in experiments with *S. aureus* ATCC 29213, lacking the *mscL* gene, with or without SaMscL overexpression, demonstrated that the natural food-borne compound xanthorrhizol targets SaMscL (Figure 3). Our results showed that single cysteine substitutions of many amino acid residues in the SaMscL protein led to a significant decrease in the antimicrobial activity of xanthorrhizol, especially in exponentially growing cells, indicating multiple binding sites for xanthorrhizol with the SaMscL protein. This may explain the rapid killing effect of xanthorrhizol against methicillin-sensitive and MRSA strains during the first 10 min of incubation with xanthorrhizol.

Xanthorrhizol enhanced the antimicrobial effect of DHS against the SaMscL channel in a MscL-dependent manner (Figure 4). A similar effect has been previously described for other antimicrobial compounds, such as 011A and K05, which enhance the effectiveness of DHS, kanamycin, tetracycline and ampicillin against *S. aureus* and *M. smegmatis*, suggesting their potential use as antibiotic adjuvants (Wray et al., 2019a, 2020). The authors hypothesized that modulation of MscL gating caused by the above-mentioned compounds may specifically permeabilize the membrane with subsequent facilitation of antibiotic uptake into the cytoplasm. These compounds have been classified as a group that directly affects mechanosensitive channels (Sidarta et al., 2022). Xanthorrhizol apparently belongs to this group, which is confirmed by the identification of amino acid residues that promote its binding to the SaMscL channel. However, a visible decrease in the viability of mutant Δ*mscL* cells after combined treatment with DHS and xanthorrhizol may indicate that SaMscL is not a unique pathway for entry into *S. aureus* cells. This assumption is supported by our findings that a mutant strain of *S. aureus* lacking the *mscL* gene remained sensitive to xanthorrhizol, but at concentrations higher than those observed for the wild-type strain (Figures 3A, 4B). Xanthorrhizol appears to share some of its antimicrobial properties with curcumin, which also limits bacterial growth in a MscL-dependent manner and increases the activity of MscL channels, possibly due to changes in biophysical properties of the membrane, for example by its thinning, since the direct binding site for curcumin has not been identified (Wray et al., 2021; Lane and Pliotas, 2023). Curcumin, a flavonoid polyphenol isolated from rhizome of turmeric (*Curcuma longa* L.) (Teow et al., 2016), which is active against both gram-negative and gram-positive bacteria, but its activity against *S. aureus* is less than that of xanthorrhizol (Teow et al., 2016; our unpublished data).

Based on the results presented in this study, we hypothesize that xanthorrhizol, an antimicrobial agent of plant origin, may be preferable to artificially synthesized compounds for antibacterial therapy. Xanthorrhizol is a multitarget antibiotic that has been evolutionarily adapted to effectively kill gram-positive bacteria, many of which are harmful to humans, but not gram-negative bacteria. The selective activity of xanthorrhizol is especially important for the human body since most gut-resident *E. coli* cells prevent colonization by pathogenic microflora and produce vitamins K and B12, which are essential for the blood coagulation process and red blood cell formation (Cepas and Soto, 2020). On the other hand, some *E. coli* species are known to cause infections of the intestines or urinary tract, which necessitates their elimination. To solve this problem, xanthorrhizol has been shown to have good antimicrobial effects against four gram-negative bacteria (*E. coli*, *Salmonella enterica* serovar Typhi, *S. enterica* serovar Typhimurium, and *Vibrio cholerae*) in combination with polymyxin B nonapeptide and some food-grade antimicrobial agents (Kim et al., 2019).

## Conclusion

In sum, we found here that the SaMscL channel is required for xanthorrhizol-dependent decrease in growth and viability of *S. aureus* cells. Treatment with xanthorrhizol increases the effectiveness of another MscL-targeting antibiotic DHS, and also results in potassium and glutamate fluxes, which is consistent with the opening of the SaMscL channel by xanthorrizol. Our findings thus demonstrate that the SaMscL protein may truly be a target for xanthorrhizol, which provides the opportunity to use this agent as a potential antibiotic against *S. aureus.* The high level of conservation of the mechanosensitive channel MscL among bacterial species can be used to further improve the function of xanthorrhizol and to develop a safe antimicrobial agent for use both against gram-positive pathogens and in combination with other agents against gram-negative pathogens.

## Data availability statement

The original contributions presented in the study are included in the article/Supplementary material, further inquiries can be directed to the corresponding author/s.

## Author contributions

EM: Formal analysis, Investigation, Methodology, Validation, Writing – original draft, Writing – review & editing. JK: Methodology, Software, Writing – original draft, Writing – review & editing. HJ: Formal analysis, Methodology, Software, Writing – review & editing. KN: Conceptualization, Writing – original draft, Writing – review & editing. J-GP: Conceptualization, Funding acquisition, Methodology, Project administration, Supervision, Writing – original draft, Writing – review & editing.
