# Supplementary figures and images for: The efficacy of the food-grade antimicrobial xanthorrhizol against Staphylococcus aureus is associated with McsL channel expression
Source: Front Microbiol. 2024 Jul 3;15:1439009. doi: 10.3389/fmicb.2024.1439009 (PMC11251944; doi:10.3389/fmicb.2024.1439009)

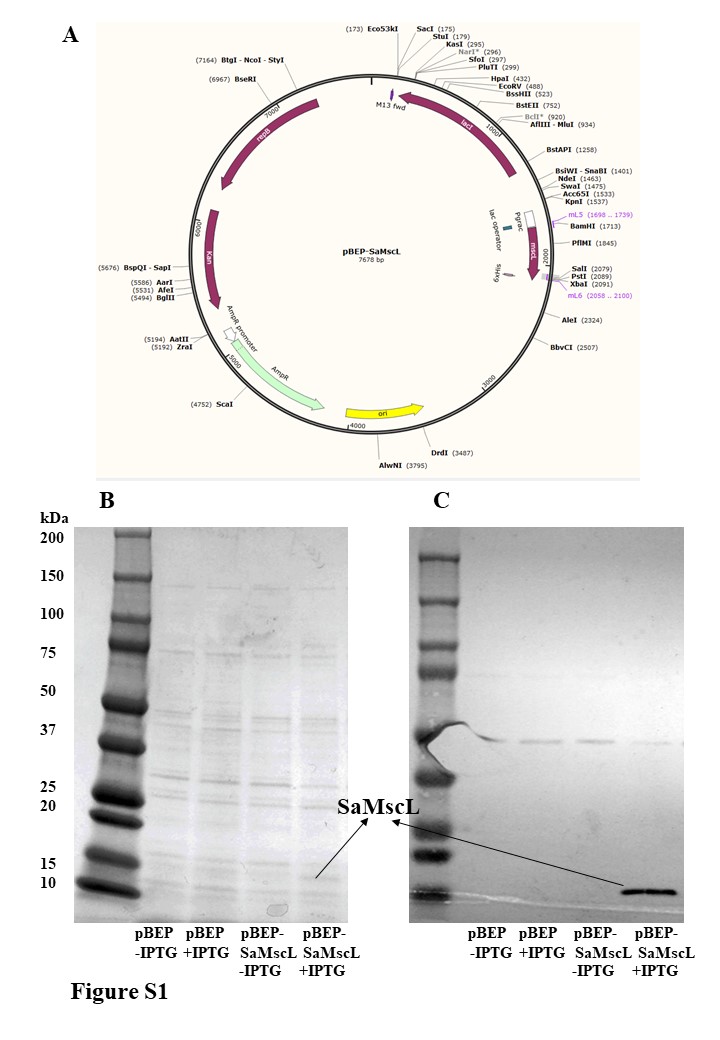

Supplement: Supplementary file 1 [file Image_1.JPEG]
