# Supplementary material for: The efficacy of the food-grade antimicrobial xanthorrhizol against Staphylococcus aureus is associated with McsL channel expression
Source: Front Microbiol. 2024 Jul 3;15:1439009. doi: 10.3389/fmicb.2024.1439009 (PMC11251944; doi:10.3389/fmicb.2024.1439009)
Supplement: Supplementary file 2 [file Data_Sheet_1.DOCX]

**Supplementary Material**

**The Efficacy of the Food-Grade Antimicrobial Xanthorrhizol against *Staphylococcus aureus* is Associated with McsL Channel Expression**

**Elena A. Mordukhova^1^, Jongwan Kim^2^, Haiyan Jin^3^, Kyoung Tai No^4^, Jae-Gu Pan^1,5^***

^1^GenoFocus Ltd., 65 Techno 1-ro, Gwanpyeong-dong, Yuseong-gu, Daejeon, 34014, Republic of Korea.

^2^Bioinformatics and Molecular Design Research Center (BMDRC), Incheon 21983, Republic of Korea

^3^The Interdisciplinary Graduate Program in Integrative Biotechnology and Translational Medicine, Yonsei University, Incheon 21983, Republic of Korea

^4^Department of Biotechnology, Yonsei University, 50-Yonsei-ro, Seodaemun-gu, Seoul, 03722, Korea

^5^Infectious Disease Research Center, Korea Research Institute of Bioscience and Biotechnology (KRIBB), 111 Gwahangno, Yuseong, Daejeon, 34141, Republic of Korea

*** Correspondence:**Corresponding Author

E-mail address: [jgpan@genofocus.com](mailto:jgpan@genofocus.com), [jgpan@kribb.re.kr](mailto:jgpan@kribb.re.kr)

**Keywords:** Food-grade antimicrobial, xanthorrhizol, MscL, *Staphylococcus aureus,* mutants.

**Table S1.** **Primer sequences used for the construction of single-site SaMscL mutants**

| **Primer** | **Mutation** | **Sequence** |
| --- | --- | --- |
| SauC4 | E4C | caattaaaggaggaaggatccatgttaaaatgcttcaaag |
| SauC5 | F5C | caattaaaggaggaaggatccatgttaaaagaatgcaaag |
| SauC8 | F8C | ggaggaaggatccatgttaaaagaattcaaagagtgcgcc |
| SauC14 | V14C | cgccttaaaaggtaactgcttagatttagc |
| SauC15 | V14C | gctaaatctaagcagttaccttttaaggcg |
| SauC23 | M23C | gcaattgctgttgtgtgcggtgcagctttcaac |
| SauC24 | M23C | gttgaaagctgcaccgcacacaacagcaattgc |
| SauC73 | F73C | caatctgttatcgactgtattatcatcgcgtttgc |
| SauC74 | F73C | gcaaacgcgatgataatacagtcgataacagattg |
| SauC76 | A77C | gttatcgactttattatcatctgctttgctttattcatc |
| SauC77 | A77C | gatgaataaagcaaagcagatgataataaagtcgataac |
| SauC78 | F78C | ctttattatcatcgcgtgtgctttattcatcttc |
| SauC79 | F78C | gaagatgaataaagcacacgcgatgataataaag |
| SauC83 | V84C | gtttgctttattcatcttctgtaagattgcaaataccttaatg |
| SauC84 | V84C | cattaaggtatttgcaatcttacagaagatgaataaagcaaac |
| SauC85 | F85C | gtttgctttattcatcttcgtttgtattgcaaataccttaatg |
| SauC86 | F85C | cattaaggtatttgcaatacaaacgaagatgaataaagcaaac |

Changes in the sequence are underlined.

**Table S2.** **Quantitative parameters of *S. aureus* ATCC 29213 cells (n=12) treated with xanthorrhizol**

|  | **Time of exposure, min** | | | | | |
| --- | --- | --- | --- | --- | --- | --- |
|  | **0** | **5** | **10** | **15** | **20** | **25** |
| **Volume (µm³)** | 2.5849±0.4745 | 2.8847±0.5536 | 3.1039±0.6051 | 2.7977±0.6051 | 2.7921±0.5710 | 2.6489±0.4926 |
| **Surface area (µm²)** | 10.8624±1.5199 | 11.6504±1.5199 | 12.2589±1.8650 | 11.3068±1.7654 | 11.2967±1.7092 | 11.0445±1.5135 |
| **Dry mass (pg)** | 0.4440±0.0771 | 0.5010±0.0867 | 0.5499±0.1028 | 0.4960±0.0990 | 0.4893±0.0990 | 0.4087±0.0793 |
| **Concentration (pg/µm³)** | 0.1724±0.0111 | 0.1746±0.0125 | 0.1780±0.0148 | 0.1786±0.0184 | 0.1764±0.0182 | 0.1550±0.0165 |
| **Mean RI** | 1.3665±0.0021 | 1.3669±0.0024 | 1.3675±0.0028 | 1.3676±0.0035 | 1.3672±0.0035 | 1.3632±0.0031 |

**Table S3.** **Quantitative parameters of *S. aureus* ATCC 29213 cells (n=11) treated with DMSO**

|  | **Time of exposure, min** | | | | | |
| --- | --- | --- | --- | --- | --- | --- |
|  | **0** | **5** | **10** | **15** | **20** | **25** |
| **Volume (µm³)** | 3.2611±0.7049 | 3.4191±0.7946 | 3.5624±0.7580 | 3.7164±0.8083 | 3.8525±0.9138 | 4.1924±1.0937 |
| **Surface area (µm²)** | 12.7968±2.0234 | 13.1618±2.2422 | 13.5007±2.1523 | 13.9366±2.2188 | 14.2623±2.4479 | 15.2443±2.9537 |
| **Dry mass (pg)** | 0.5131±0.1385 | 0.5466±0.1539 | 0.5665±0.1567 | 0.5892±0.1654 | 0.6038±0.1728 | 0.6634±0.1917 |
| **Concentration (pg/µm³)** | 0.1569±0.0131 | 0.1594±0.0113 | 0.1587±0.0152 | 0.1581±0.0164 | 0.1562±0.0148 | 0.1587±0.0164 |
| **Mean RI** | 1.3635±0.0025 | 1.3640±0.0022 | 1.3638±0.0029 | 1.3637±0.0031 | 1.3634±0.0028 | 1.3638±0.0031 |

**Figure legend**

**Supplementary Figure 1.** **Map of the pBEP-SaMscL plasmid (A) and IPTG-induced SaMscL protein expression in *S. aureus* ATCC 29213Δ*mscL* cells.**

Exponentially growing cultures of *S. aureus* ATCC 29213Δ*mscL* carrying the empty expression vector pBEP or pBEP-SaMscL in TSBKan_25_ medium were induced with 1 mM IPTG for 4 h. Whole-cell lysates were subjected to 12% SDS‒PAGE (B) followed by Western blotting using Histidine (C-term) tag (6XHis) Mouse Monoclonal Antibody (C).
